# Non-corrosive Green Lubricant With Dissolved Lignin in Ionic Liquids Behave as Ideal Lubricants for Steel-DLC Applications

**DOI:** 10.3389/fchem.2019.00857

**Published:** 2019-12-06

**Authors:** Jing Hua, Yijun Shi

**Affiliations:** Division of Machine Elements, Luleå University of Technology, Luleå, Sweden

**Keywords:** lubricants, ionic liquids, ILs, lignin, DLC

## Abstract

Diamond-like carbon (DLC)–steel contacts become more and more popular in the industry now. Since the surface chemical properties of DLC are quite different from those of iron, traditional formulated lubricants have problems to form tribo-chemical films, which are effective to improve the tribological performance for steel-steel contacts, on the surface of DLC. Thus, new lubricants formulation strategies are needed to be considered for steel-DLC applications. A kind of green lubricant (lignin-[Choline][L-Proline] (L-[CH][Pro])) without any traditional tribo-chemical active element, i.e., free of P, S, B, etc., was studied in this paper for the steel-DLC contact. To find the difference between this new ILs and the traditional lubricants, a commercially available fully formulated lubricant was used as a reference. An Optimol SRV-III oscillating friction and wear tester was used to evaluate the tribological performance. Three different kinds of commercially available DLC coatings (Tribobond 40(Cr + a-C:H:W), Tribobond 43 [(Cr+) a-C:H), and Tribobond 44(a-C:Cr)] were investigated. The results show that the ILs exhibit an obviously lower friction coefficient than that of the traditional commercially available fully formulated lubricant. Among those three DLC coatings, the (Cr+) a-C:H DLC coating exhibits the biggest improvement of wear resistance lubricated with the new ILs than that of the commercially available fully formulated lubricant. It's expected that its excellent tribological properties are attributed to the affinity of the ILs to the metal surface and the strength of the ionic liquids interactions by hydrogen bonding. Thus, forming strong physical adsorption strategy, instead of forming chemical tribo-films, is recommended to enhance the lubricating performance of lubricants for DLC.

## Introduction

Diamond-like carbon(DLC) coatings are widely recognized as being excellent self-lubrication material in biomedical, automotive components and magnetic due to its low friction and high wear resistance properties (Liu et al., 1996; Donnet, 1998). The earliest study of DLC films can be traced back to 1953, and people started applying DLC in the industry in the late 1990s, i.e., applied DLC film on razor blades and fuel injector systems of diesel engines extensively (Erdemir and Donnet, 2006). Nowadays, various DLC coatings have been applied in industries to increase the working efficiency and life of machinery (Beake et al., 2015; Kim and Kim, 2015). The excellent tribological performance of DLC coatings is owing to the mixture of sp3 and sp2 hybridized carbon, and these tribological properties can be improved by alloying the DLC coatings with certain elements such as hydrogen, nitrogen, silicon, titanium, boron, and fluorine (Robertson, 2008).

Considering that the tribological behavior of the contact will be determined by chemical interactions between the oil additives, oxygen in the ambient atmosphere, and the metallic surfaces (Podgornik et al., 2003b), some researchers have already studied whether DLC is compatible with existing industrial lubricants (Podgornik et al., 2003a, 2005; Haque et al., 2007a,b; Austin et al., 2015; Qu et al., 2015). Many of these studies reported that no stable tribofilm was detected on the DLC surface when lubricated with commercially available fully formulated oil (Podgornik et al., 2003a, 2005; Haque et al., 2007a). Since results published on DLC/lubricant interactions were quite contradictory and no clear understood, there will be huge risks of damage to the contact surfaces. However, one question still remains that whether we could find better lubricants by new formulation strategies, such as using physical adsorption to form the tribofilms instead of tribo-chemical films.

Ionic liquids (ILs) have excellent anti-wear capability due to their high polarity, which can form very strong effective adsorption film (Minami, 2009; Zhou et al., 2009; Somers et al., 2013). However, traditional ILs have some disadvantages include high cost, potential toxicity, poor biodegradability and corrosivity especially those with imidazolium or pyridinium cations and halogen anions (Mu et al., 2015). Some researchers, who already have paid attention on the tribological performance lubricated by ILs, find some new ILs, such as 1-Buty-1-Methylpyrrolidinium tris(pentafluoroethyl)trifluorophosphate([BMP][FAP]), ethyl-dimethyl- 2-methoxyethylammonium tris(pentafluoroethyl)trifluoropho- sphate [(NEMM)MOE][FAP], 1-butyl-3-methylimidazolium tricyanomethanide ([BMIM] [TCC]) and 1-ethyl-3-methylimidazolium dicyanamide ([EMIM] [DCN]) (González et al., 2010, 2013; Kondo et al., 2013), which can avoid corrosive damage and achieve good lubricity. [Choline][Amino acid]([CH][AA]) ILs, which were found generally to have low toxicity and to be readily biodegradable (Hou et al., 2013), draw our attention. It could be used as environmentally friendly lubricant (Jiang et al., 2018; Wu et al., 2018; Zhang et al., 2018). Recently, we developed a green lubricant by using [CH][AA] ILs as the lubricant base and strengthened by lignin through reciprocal hydrogen bonding in between (Mu et al., 2015). The addition of lignin in [CH][AA] improves anti-wear properties and friction stability. The excellent tribological performance of L-[CH][AA] is most likely attributed to the formation of IL films by physical adsorption during the friction process, which may play a similar role for lubrication as that of tribo-chemical films.

In this work, three DLC coatings, which are widely applied in automotive components, containing two kinds amorphous hydrogenated DLC coatings and one kind of H-free DLC coating are studied. The tribological performance of these DLC coatings lubricated with L-[CH][AA] is investigated and compared with that of a commercially available fully formulated lubricant.

## Materials and Methods

### Materials

**Figure 1:**
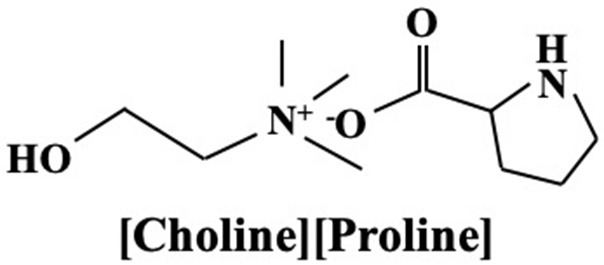
Molecular structures of [Choline][L-Proline].

The [CH][AA] used in this study is [Choline][L-proline] ([CH][Pro]), the molecular structure of which is shown in Figure 1. 7 wt% lignin was added into the ILs for enhancing the lubricating performance, which is used as the lubricant (L-[CH][Pro]) in this paper. More details about the synthesis of L-[CH][Pro] can be found in our previous work (Mu et al., 2015). Gearway S5 75w-140, denoted GW/S5, was supplied by Statoil Fuel & Retail Lubricants Sweden AB.

The DLC coatings used in this work were supplied by IonBond^1^ Sweden AB. Three types of DLC coatings: Tribobond 40(Cr + a-C:H:W), which is an amorphous hydrogen-terminated DLC doped with W and uses Cr behaves as the bonding layer, Tribobond 43 ((Cr+) a-C:H), which is an amorphous hydrogen-terminated DLC and uses Cr behaves as the bonding layer, Tribobond 44(a-C:Cr), which is an amorphous DLC and uses Cr behaves as the bonding layer, were investigated in this paper. Table 1 shows the characteristic of these three DLC coatings. All of them are well-proven DLC coatings for tools, industrial and automotive components. Specifically, Tribobond 40 (Cr + a-C:H:W) has good impact fatigue resistance and is suitable for contacts in rolling motions due to its ductile behavior. The major advantage of Tribobond 43 ((Cr+) a-C:H) is absolute chemical inertness, making it non-sensitive to any environmental influence. Tribobond 44 (a-C:Cr) can offer good impact fatigue resistance at elevated temperatures.

**Table 1:** Physical properties of DLC coatings.

**Coating**	**Material**	**Thickness range, μm**	**Micro hardness** **HV 0.05**	**Color**
Tribobond 40	Cr+ a-C:H-W	1–10	1,000–1,800	Gray
Tribobond 43	(Cr+) a-C:H	1–5	2,500–4,000	Black
Tribobond 44	a-C:Cr	1–5	1,000–1,400	Gray

### Experiment

The lubricity of the L-[CH][Pro] and GW/S5 was investigated using an Optimol SRV-III oscillating friction and wear tester. The upper steel ball (52100 bearing steel, diameter 10 mm, surface roughness (Ra) 20 nm, provided by SKF, Sweden) slides under reciprocating motion against tested disks. The tested disks consist of one steel disc (100CR6 ESU hardened, Ø24 mm 7.9 mm, and surface roughness (Ra) 120 nm, supplied by Optimol Instruments Prüechnik GmbH, Germany) and three disks within DLC coatings (supplied by IonBond company, Sweden). Before each test the device and specimens were cleaned with acetone and ethanol. Tests of were conducted under the load of 33N (1.51 GPa maximum Hertzian pressure) and 77N (2 GPa maximum Hertzian pressure) at room temperature (25°C), a sliding frequency of 50 Hz, and an amplitude of 1 mm. A data acquiring system linked to the SRV-III tester was used to record the friction coefficient curves automatically. Three duplicate friction and wear tests were carried out to minimize experimental error. After the tests, 3D topography of the disc surfaces and ball surfaces were determined using an optical system (Zygo 7300). The elements and their distributions on the worn surfaces were measured by SEM (Merlin FEG-SEM) with an energy dispersive spectroscopic (EDS) detector. The elastic modulus and nanoindentation hardness of Tribobond 40 (Cr + a-C:H:W) coating were directly measured by nanoindenter (Micro Materials NanoTest Vantage system) in a load- controlled manner with a maximum load of 1 mN. The relative humidity in the lab was ~20%.

## Results

### Friction

**Figure 2:**
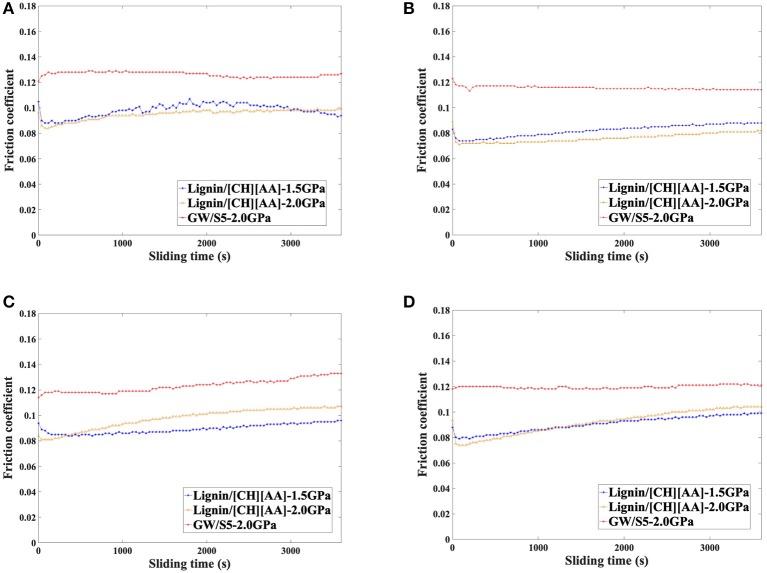
Friction behavior of each disc: **(A)** steel disc, **(B)** Tribobond 40 disc, **(C)** Tribobond 43 disc, **(D)** Tribobond 44 disc.

Figure 2 shows the friction coefficient evolution during 1 h friction test with the presence of L-[CH][Pro] and GW/S5 under the load of 33N (1.51 GPa maximum Hertzian pressure) and 77N (2 GPa maximum Hertzian pressure) at room temperature (25°C). In the case of using L-[CH][Pro] as lubricants, it seems that the pressure has very little effect on the friction coefficient of all the materials tested in this study.

All the specimens lubricated with L-[CH][Pro] show a lower friction coefficient than those lubricated with GW/S5. Compared with GW/S5, widely used in the vehicle industry, it looks like that the L-[CH][Pro] exhibits better lubricity with all the materials studied in this work.

### Wear of Disc

**Figure 3:**
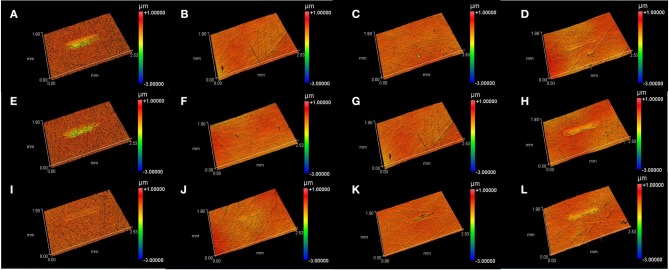
3D microscopic images of wear tracks on disc surfaces: **(A)** steel disc lubricated with L-[CH][Pro]-1.5GPa, **(B)** Tribobond 40 DLC lubricated with L-[CH][Pro]-1.5GPa, **(C)** Tribobond 43 DLC lubricated with L-[CH][Pro]-1.5GPa, **(D)** Tribobond 44 DLC lubricated with L-[CH][Pro]-1.5GPa, **(E)** steel disc lubricated with L-[CH][Pro]-2.0GPa, **(F)** Tribobond 40 DLC lubricated with L-[CH][Pro]-2.0GPa, **(G)** Tribobond 43 DLC lubricated with L-[CH][Pro]-2.0GPa, **(H)** Tribobond 44 DLC lubricated with L-[CH][Pro]-2.0GPa, **(I)** steel disc lubricated with GW/S5-2.0GPa, **(J)** Tribobond 40 DLC lubricated with GW/S5-2.0GPa, **(K)** Tribobond 43 DLC lubricated with GW/S5-2.0GPa, **(L)** Tribobond 44 DLC lubricated with GW/S5-2.0GPa.

**Figure 4:**
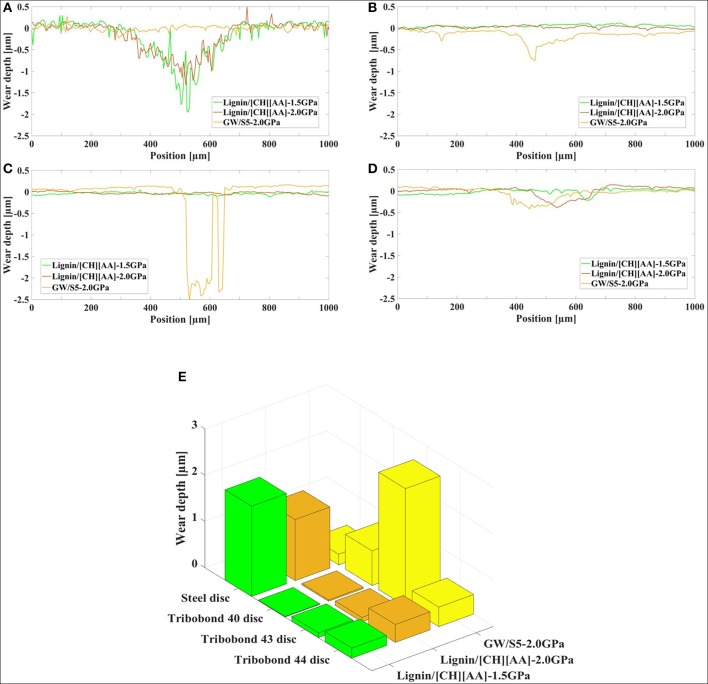
Cross-sectional profile of typical worn surface: **(A)** steel disc, **(B)** Tribobond 40 disc, **(C)** Tribobond 43 disc, **(D)** Tribobond 44 disc; **(E)** Comparison of wear depth.

Figure 3 presents the 3D topography of the disc surface lubricated by L-[CH][Pro] and GW/S5 after the test, and Figure 4 gives the cross-sectional profile of typical worn surface of disc tested in this study. We find that the steel surface lubricated by L-[CH][Pro] shows remarkable difference in contrast to that lubricated by GW/S5, and GW/S5 significantly reduces the width and depth of the worn tracks in Figures 3I, 4A. The steel disks lubricated with L-[CH][Pro] shows severe wear damage, as it can be observed from Figures 3A,E, 4A.

On the other hand, it can be observed that the disc with Tribobond 40(Cr + a-C:H:W) lubricated by L-[CH][Pro] has negligible wear scarring on the sliding surface (Figures 3B,F), whereas the surface lubricated by GW/S5 shows small and narrow wear tracks (Figure 3J). From Figures 3C,G,K, it can be seen that tests made with Tribobond 43 ((Cr+) a-C:H) coatings present similar results to those coated with Tribobond 40. And, as shown in Figures 4C,E, it's worth noting that the wear depth of Tribobond 43 lubricated with GW/S5 was significantly deeper than those lubricated with L-[CH][Pro]. In contrast with amorphous hydrogenated DLC coatings, the surfaces of Tribobond 44(a-C:Cr) lubricated with L-[CH][Pro] and GW/S5 both show larger and deeper wear tracks(Figures 3D,H,I).

### Wear of Ball

**Figure 5:**
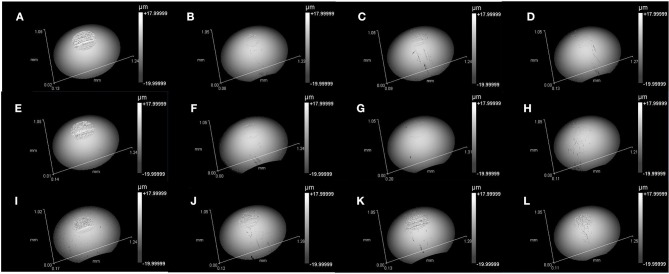
3D microscopic images of wear tracks on ball surfaces: **(A)** steel disc lubricated with L-[CH][Pro]-1.5GPa, **(B)** Tribobond 40 DLC lubricated with L-[CH][Pro]-1.5GPa, **(C)** Tribobond 43 DLC lubricated with L-[CH][Pro]-1.5GPa, **(D)** Tribobond 44 DLC lubricated with L-[CH][Pro]-1.5GPa, **(E)** steel disc lubricated with L-[CH][Pro]-2.0GPa, **(F)** Tribobond 40 DLC lubricated with L-[CH][Pro]-2.0GPa, **(G)** Tribobond 43 DLC lubricated with L-[CH][Pro]-2.0GPa, **(H)** Tribobond 44 DLC lubricated with L-[CH][Pro]-2.0GPa, **(I)** steel disc lubricated with GW/S5-2.0GPa, **(J)** Tribobond 40 DLC lubricated with GW/S5-2.0GPa, **(K)** Tribobond 43 DLC lubricated with GW/S5-2.0GPa, **(L)** Tribobond 44 DLC lubricated with GW/S5-2.0GPa.

**Figure 6:**
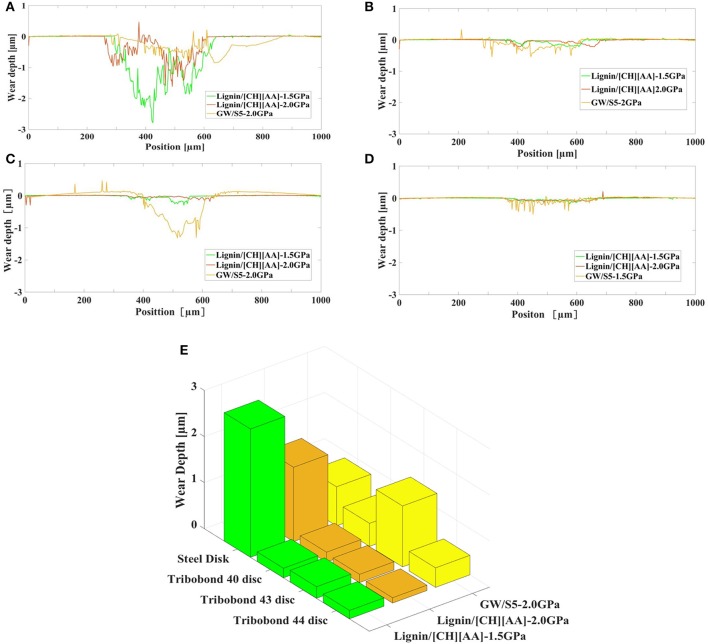
Cross-sectional profile of typical worn surface of steel ball: **(A)** steel disc, **(B)** Tribobond 40 disc, **(C)** Tribobond 43 disc, **(D)** Tribobond 44 disc; **(E)** Comparison of wear depth.

The 3D microscopic images of the corresponding wear scars on the steel balls are provided in Figure 5. It can be observed that big wear scars always appear in steel/steel contacts from Figures 5A,E,I. Figure 6 presents the typical cross-sectional profiles on the worn ball surfaces, in which the software provided from Zygo was used to remove the sphere shape into a plate shape for us to be possible to evaluate the wear depth of the upper steel.

It is obvious that using L-[CH][Pro] as a lubricant for DLC coatings surface can not only protect the disc surfaces, but also reduce the wear on the steel balls. As shown in Figures 6B,C,D,E, the wear depth of upper steel balls for DLC coatings lubricated with L-[CH][Pro] was significantly lower than those lubricated with GW/S5. It's rather remarkable that the wear tracks on the Tribobond 44(a-C:Cr) coating surface are larger and deeper than all the other specimens. No obvious wear of the upper steel ball can be found for all cases of DLC coatings lubricated by L-[CH][Pro]. Instead, only a slight roughness reduction on the surfaces can be observed (Figures 5D,H, 6D).

## Discussion

It is well-known that the anti-wear additives such as Zinc dialkyldithiophosphate (ZDDP) are widely used in lubricated systems, they can form a chemical tribofilm on steel surface to reduce wear (Willermet et al., 1995). In this study we have found that GW/S5 significantly reduces the wear for steel/steel contacts (Figure 4A), but it does not work for steel/DLC contacts. Herein, from the EDS Elemental wt% analysis (Table 2) on the worn surface of disks, we can find the tribo-chemical active element, S and P signature when SW/S5 is used as lubricant for steel/steel, which indicates that the good anti-wear behavior of the traditional formulated lubricants is due to the formed chemical tribofilm, containing S and P elements.

**Table 2:** Elemental wt% of EDS study on the worn surface of disks.

**Spectrum**	**Fe**	**S**	**P**	**O**	**C**	**N**	**W**	**Cr**	**Si**
steel disc before sliding	93.1			1.58	3.82	0.07		0.3	1.13
Steel/Steel, SW/S5	83.6	1.72	2.01	7.89	4.14	0.64			
Steel/Steel, L-[CH][Pro]	91.57			1.9	3.76			1.54	1.23
Steel/Tribobond 40 before sliding	3.53			1.6	28	0.09	63.34	3.44	
Steel/Tribobond 40, SW/S5	1.48		0.32	0.57	23.19		74.44		
Steel/Tribobond 40, L-[CH][Pro]	5.21			0.93	21	0.05	69.43	3.38	

On the contrary, the traditional formulated lubricants have some problems with steel/DLC contacts. One significant experiment phenomenon worth noting is both the wear of the disc and the upper steel ball in the case of Tribobond 43 ((Cr+) a-C:H) lubricated with GW/S5 is the biggest (Figures 4C, 6C), while that L-[CH][Pro] gives an obvious much lower wear. The worst anti-wear ability of GW/S5 for steel/DLC can be attribute the phosphate additives existed in the traditional formulated. Similar phenomena was reported by Zhou et al. (2015). It is believed that the phosphate additives increased wear rate when a steel ball rubbed against a DLC coating. In our EDS study, P signature is also found in the case of steel/DLC lubricate by SW-75. The carbon in DLC catalyzes the tribochemical reactions between the phosphate additives and the steel ball surface, leading synergistic tribochemical material removal, or tribo-corrosion.

On the other hand, L-[CH][Pro] lubricates very well for steel/DLC contacts. For example, the disks with Tribobond 40 (Cr + a-C:H:W) and Tribobond 43 ((Cr+) a-C:H) DLC coatings lubricated by L-[CH][Pro] have negligible wear scars. All the balls for steel/DLC contacts lubricated by L-[CH][Pro] have almost no wear. Normally, the tribological properties of DLC coatings rely on their structural and chemical nature, humidity, temperature, working conditions, surrounding environment, substrate materials, and counterpart materials (Hassan et al., 2013). In our experiment, we can conclude that the only H-free DLC coating (a-C:Cr) would have large and deep wear tracks on its surface, while the other two kinds of amorphous hydrogenated DLC coatings have negligible wear scarring on the sliding surface. The similar phenomenon can be found in some other researchers' experiments under lubricated condition, Austin et al. reported that the a-C:H DLC coatings performed better and showed less wear than the Si-DLC and W-DLC coatings in fully formulated oil+ZDDP (Austin et al., 2015); Kondo et al. (2013) revealed that the H-free DLC coating lubricated with [EMIM][TCC] showed severe wear damage. The mechanism of better anti-wear performance of H-DLC was discussed by Hsu et al. (2008), and which was attributed that the increased hydrogen concentration causes the decrease of sp3 ration, and modulus.

To further study the mechanism of the excellent tribological performance of L-[CH][Pro] for H-DLC, the hardness and elastic modulus of Tribobond 40(Cr + a-C:H:W) coating both before and after friction test were tested by nanoindenter, shown in Table 3. The difference of two H-DLC coatings chosen in this work is that Tribobond 43 ((Cr+) a-C:H) has the characteristic of absolute chemical inertness. Thus, we chose a more tribochemical active H-DLC [Tribobond 40 (Cr + a-C:H:W)] to study the difference of hardness and elastic modulus before and after sliding. Twelve measurements were carried out for each surface to minimize experimental error and the average hardness, elastic modulus and elastic recovery were calculated based on these. Comparing with experimental error, the changes of these mechanical properties are not impressive. The elastic recovery before and after friction test is almost same, thus the mechanism of changed mechanical properties caused by tribochemical transfer film is not very suitable to explain the excellent tribological of L-[CH][Pro] for steel/DLC. In our previous work, we reported that the overall tribological performance of L-[CH][Pro] was determined by the affinity of the ionic liquid to the metal surface and the strength of the ionic liquids-lignin interactions by hydrogen bonding (Mu et al., 2015). Similarly, in another previous work, we find that strong physical adsorption of ionic grease onto friction surface plays a dominating role for promoted lubrication instead of tribo-chemical film formation (Mu et al., 2016). This theory could be used for explaining L-[CH][Pro] exhibits overall tribological performance than that of GW/S5 for steel-DLC contacts. [CH][Pro] ILs are most likely attributed to the formation of IL films by physical adsorption during the friction process, it prevents direct contact between the steel ball and DLC coatings to reduce the friction and wear loss; the reciprocal hydrogen bonds between lignin and [CH][Pro] help to improve the mechanical strength of the lubrication film and result in effective interfacial separation between the upper steel ball and DLC coatings.

**Table 3:** The mechanical properties of Tribobond 40 DLC coating surfaces lubricated by L-[CH][Pro].

	**Before friction test**	**After friction test**
Elastic modulus (GPa)	162 ± 27	154 ± 20
Nanoindentation hardness (GPa)	13.4 ± 3.4	12.5 ± 2.8
Elastic recovery	0.42 ± 0.07	0.41 ± 0.08

## Conclusions

The tribological properties of L-[CH][Pro] for DLC coatings were investigated in the present study. The following conclusions can be made from this study:

1. The presence of the L-[CH][Pro] results in a friction reduction for all the cases compared with commercially available full formulated lubricants.
2. Tribological tests using L-[CH][Pro] as lubricant in the case of amorphous hydrogenated DLC coatings showed the lower friction and lower wear loss. only negligible wear scars appear in the sliding surfaces in these cases.
3. L-[CH][Pro] can not only provide a good protection of the DLC coatings, but also can protect the upper steel ball very well.
4. The strategy of using physical adsorption to form the tribo-films instead of forming tribo-chemical films is suitable for steel-DLC contacts.

## Data Availability Statement

All datasets generated for this study are included in the article/supplementary material.

## Author Contributions

JH designed and performed experiments under supervision of YS. JH wrote the paper. YS revised the paper.

### Conflict of Interest

The authors declare that the research was conducted in the absence of any commercial or financial relationships that could be construed as a potential conflict of interest.

## Footnotes

^1^CAS. *Ionbond—Coating Portfolio*. Available at: https://www.ionbond.com/coating-services/automotive/coating-portfolio/; http://www.cs2.ch
